# Analysis of HIV-1 envelope evolution suggests antibody-mediated selection of common epitopes among Chinese former plasma donors from a narrow-source outbreak

**DOI:** 10.1038/s41598-018-23913-2

**Published:** 2018-04-10

**Authors:** Sophie M. Andrews, Yonghong Zhang, Tao Dong, Sarah L. Rowland-Jones, Sunetra Gupta, Joakim Esbjörnsson

**Affiliations:** 1 Nuffield Department of Medicine, University of Oxford, Oxford, United Kingdom; 2 Beijing You’an Hospital, Capital Medical University, Beijing, China; 3 Weatherall Institute of Molecular Medicine, University of Oxford, Oxford, United Kingdom; 4 Department of Zoology, University of Oxford, Oxford, United Kingdom; 5 Department of Laboratory Medicine, Lund University, Lund, Sweden

## Abstract

The HIV-1 envelope mutates rapidly to evade recognition and killing, and is a major target of humoral immune responses and vaccine development. Identification of common epitopes for vaccine development have been complicated by genetic variation on both virus and host levels. We studied HIV-1 envelope *gp120* evolution in 12 Chinese former plasma donors infected with a purportedly single founder virus, with the aim of identifying common antibody epitopes under immune selection. We found five amino acid sites under significant positive selection in ≥50% of the study participants, and 22 sites consistent with antibody-mediated selection. Despite strong selection pressure, some sites housed a limited repertoire of amino acids. Structural modelling revealed that most of the variable amino acid sites were located on the exposed distal edge of the Gp120 trimer, whilst invariant sites clustered within the centre of the protein complex. Two sites, flanking the V3 hypervariable loop, represent novel antibody sites. Analysis of HIV-1 evolution in hosts infected with a narrow-source virus may provide insight and novel understanding of common epitopes under antibody-mediated selection. If verified in functional studies, such epitopes could be suitable as targets in vaccine development.

## Introduction

The human immunodeficiency virus type 1 (HIV-1) glycoprotein Gp120 is a 120 kDa surface-expressed protein that is essential for viral entry into the cell. It is encoded by the *env* gene, and consists of five variable regions (V1-V5) interspersed between five conserved regions (C1-C5)^1^. The Gp120 forms heterodimers with Gp41 which themselves trimerise, studding the viral membrane at a density of around fourteen copies per virion^2^. Whilst the cellular immune response against HIV-1 targets epitopes dispersed throughout the viral genome, the accessibility of Gp120 on the cell surface makes it the major target of humoral responses and development of HIV vaccines and antibody-based immunotherapy.

The humoral response against HIV-1 Gp120 develops rapidly within around four weeks of detectable plasma viral loads^3^, but neutralising antibodies (NAbs) typically only develop after several months of infection^4^. Around two hundred antibodies have been described that recognise the Gp120 protein (LANL Immunology Database; http://www.hiv.lanl.gov/content/immunology), and many of the epitopes cluster within the V3 loop. However, the interplay between Gp120 and the adaptive immune response is complex, and the role that antibodies play in the control of infection is a contentious issue. Studies in macaques have indicated that B lymphocyte depletion-associated reductions in NAb titre inversely correlate with viral load, suggesting that the humoral response may contribute at least in part to the control of viral replication^5,6^. In addition, the loss of neutralising activity has been associated with faster disease progression in some individuals^7^. However, whilst NAbs do exert selection pressure on the virus^8,9^, the breadth of response does not correlate with or predict progression to AIDS^7,10,11^.

It is commonly believed that the reason why antibody responses may play a limited role in the control of HIV-1 is because the virus can mutate easily to escape neutralisation by these responses: as one antibody is evaded, new antibodies arise and are evaded in a continuous cycle^9,12–14^. This view is supported by the observation that HIV-1 is rarely susceptible to neutralisation by contemporaneous antibodies in early infection^15,16^, whilst the same antibodies are able to effectively neutralise historic virus^9,12,17^. However, in clinically latent infection, viral variants evolve susceptibility to neutralisation by contemporaneous NAbs, or to sera sampled much earlier in infection^18–20^. It is therefore possible that antibody responses do play an important role in controlling HIV-1, at least in the latent phase, with re-emergence of variants occurring periodically as the associated NAb responses fall below a certain threshold but then are restored by stimulation by the variant^21^.

Indeed, several apparent paradoxes in HIV-1 pathogenesis and the genetics of host susceptibility can be resolved by assuming that NAbs play an important role in the control of infection, as shown by a recent modelling study^21^. Non-neutralising responses with Fc-related activities – including antibody-dependent cellular cytotoxicity (ADCC) or antibody-mediated cellular viral inhibition (ADCVI) – directed at epitopes of intermediate variability, may also help maintain chronicity of infection. This is consistent with the findings of studies in rhesus macaques demonstrating that simian immunodeficiency virus isolated during clinically latent infection remains susceptible to ADCVI responses from earlier plasma, despite no detectable contemporaneous, autologous neutralising response^22^. A potential therapeutic approach to preventing disease progression may therefore be to develop vaccines that boost and maintain such partially cross-protective responses.

HIV-1 is one of the fastest evolving organisms known to science due to extremely high mutation, recombination and replication rates^23^. This leads to vast genetic diversity, and HIV-1 variants can differ genetically by >5% in an infected individual at a single time-point. The transmission of HIV-1 is associated with a major bottleneck, and in most cases, new infections are the result of the outgrowth of one single transmitted founder virus^24,25^. During infection, HIV-1 continues to evolve at a high rate with diversification driven to a large extent by adaptive immune responses^26–31^. Mutations that facilitate immune evasion are positively selected and becomes dominant in the viral population^32^. Several studies have also shown a positive correlation between HIV-1 evolutionary rate and disease progression^33–37^.

Here we studied *gp120* evolution in a narrow-source cohort of former plasma donors (FPDs) from Henan Province in China (SM cohort)^38^. The FPDs of Henan were infected with a narrow-source of virus through exposure to contaminated equipment, and transfusion with pooled red blood cells during an illegal paid plasma donation scheme in the mid-1990s^39,40^. Owing to the unusually homogeneous route and source of infection, and the narrow time frame during which study participants were exposed, the infecting founder was relatively conserved. To our knowledge, this is the first in-depth longitudinal study of HIV-1 envelope evolution in a population infected from a single source. This provided us with the unique opportunity to investigate where and how immunological selection pressure drives mutation within *gp120*. Our aim was not only to comprehensively map and identify potential antibody-restricted epitopes in natural infection, but also to understand the constraints placed on substitution in these regions, thereby testing whether positive selection exhibited fluctuating or consistent patterns of immune escape over time. The identification of such epitopes of limited variability, which are nonetheless targets of natural immunity, may assist in the development of vaccination strategies that prevent viral escape: for example, by incorporating all possible substitution forms into the vaccine.

## Results

### Cohort characteristics

HIV-1 *env gp120* sequences were recovered from 12 study participants in the SM cohort (Table 1). The HLA types of those were representative of cohort frequencies^38^. Sequence recovery was 74% from the available specimens (Fig. S1A). PCR success was associated with the viral load of the sample. The final dataset consisted of 575 sequences. Across the specimens sampled, median viral load was 7,388 copies ml^−1^ of plasma (interquartile range (IQR): 1,612-30,403); median absolute CD4^+^ lymphocyte count was 337 cells µl^−1^ (IQR: 248–400); and median CD4 percentage of lymphocytes was 24% (IQR: 15–30). Demographic and clinical characteristics of the study participants sampled are shown in Table 1.

**Table 1:** Demographic and clinical characteristics of the SM cohort study participants sampled.

Patient	Sex^1^	HLA Type						2010				2011				2012				2014			
		A1	A2	B1	B2	C1	C2	VL^2^	CD4 abs^3^	CD4%^4^	ART^5^	VL^2^	CD4 abs^3^	CD4%^4^	ART^5^	VL^2^	CD4 abs^3^	CD4%^4^	ART^5^	VL^2^	CD4 abs^3^	CD4%^4^	ART^5^
SM007	F	1	2	40	57	6	8	7388	362	23	N	1445	300	24	N	2884	253	16	N	8733	253	15	Y
SM021	F	2	29	44	38	4	7	35827	266	18	N	N/R^6^	N/R^6^	N/R^6^	—	14782	206	20	N	N/R^6^	N/R^6^	N/R^6^	N/R^6^
SM039	F	2	11	49	39	7	7	1612	492	30	N	791	346	28	N	1732	319	18	Y	N/R^6^	N/R^6^	N/R^6^	N/R^6^
SM176	F	11	32	38	51	7	14	43569	429	30	N	435669	N/R^6^	N/R^6^	N	27348	95	3	N	50	495	28	Y
SM209	M	24	31	51	54	1	14	44584	334	33	N	1195	354	32	N	4034	478	22	N	11922	242	14	Y
SM335	M	30	31	13	40	6	3	N/R^6^	294	36	N	7751	257	33	N	71263	228	16	Y	88620	59	6	Y
SM358	F	2	11	49	50	3	6	50	469	25	N	1025	390	27	N	1716	387	25	N	N/R^6^	N/R^6^	N/R^6^	N/R^6^
SM446	M	2	26	38	51	12	14	N/R^6^	255	27	N	7226	374	31	N	3217	187	25	N	N/R^6^	N/R^6^	N/R^6^	N/R^6^
SM505	M	11	2	35	52	12	12	8871	478	19	N	175519	337	30	N	210735	402	24	N	180	593	30	Y
SM514	F	2	31	40	40	3	15	30403	389	11	N	N/R^5^	1233	66	N	5166	216	7	N	N/R^6^	N/R^6^	N/R^6^	N/R^6^
SM536	F	11	30	13	15	6	7	4812	567	31	N	23956	397	24	N	12637	351	13	N	N/R^6^	N/R^6^	N/R^6^	N/R^6^
SM538	M	2	24	13	40	3	3	409157	92	10	N	50	73	12	N	50	52	9	Y	N/R^6^	N/R^6^	N/R^6^	N/R^6^

^1^Sex: F = Female; M = Male.

^2^VL = Viral load in RNA copies per ml.

^3^CD4 abs = CD4 count in cells per µl.

^4^CD4% = CD4 cells as percentage of lymphocytes.

^5^ART = On antiretroviral treatment: Y = Yes; N = No.

^6^N/R = Data not recorded or available.

Maximum likelihood phylogenetic analysis showed a star-like relationship between the sequences, with short internal branches between study participants, consistent with a narrow-source outbreak (Fig. S1B). Subtype analysis showed that all study participant sequences clustered with the CRF15_01B strain: a circulating recombinant virus initially reported in Thailand composed of CRF01_AE with the majority of envelope being subtype B (Fig. S2). Bayesian phylogenetic analysis placed the origin of the SM cohort cluster at around January 1999 (95% HPD October 1988 to November 2006).

### Five positions in Gp120 were under significant positive selection pressure in at least half of the study participants irrespective of HLA profile

**Figure 1:**
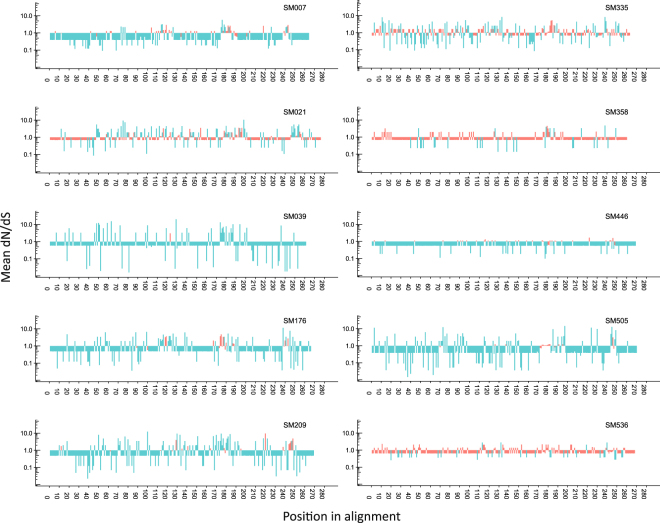
Patient-specific selection pressure within the HIV-1 envelope Gp120 protein. Ten of the 12 study participants yielded sequences from two or more time-points and were subjected to evolutionary analyses (Fig. S1A). All time-points with available sequences for the ten study participants were included in the analyses. Mean dN/dS ratios for each codon within each study participant. A dN/dS estimate greater than 1 indicates positive selection whilst an estimate of less than 1 indicates negative selection. Sites under significant selection shown in blue whilst sites that have not reached statistical significance are shown in red and are assumed neutral. Differences in the number of codons across study participants are the result of length variation in the V4 and V5 hypervariable loops.

Ten of the 12 study participants yielded sequences from two or more time-points and were subjected to evolutionary analyses (Fig. S1A). All time-points with available sequences for the ten study participants were included in the analyses. The median evolutionary rate ratio of positions 1 + 2 to position 3 among the participants was 0.861 (IQR: 0.779-0.892), indicating a general purifying selection over the HIV-1 *env gp120* C2-V5 region in the majority of the study participants (the overall ratio was <1 in all study participants except SM021 [1.306]). Next, we evaluated the intrapatient dN/dS ratio of each codon by renaissance counting^41^. The majority of codons in Gp120 were under either significant positive or negative selection in seven of the ten study participants with longitudinal samples (Fig. 1), and neutral evolution was comparatively rare. Consistent with the 1 + 2:3 codon rate ratios, only one study participant (SM021) appeared to have more residues under significant positive than negative selection pressure.

**Figure 2:**
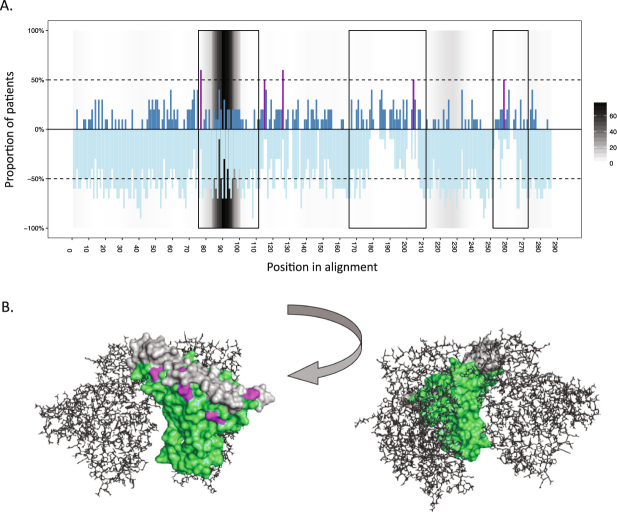
Summary of the selection pressure within the HIV-1 envelope Gp120 protein among the study participants. (**A**) The proportion (absolute) of study participants showing evidence of significant positive or negative selection across each aligned codon of the amplicon. Positive selection is shown in dark blue, whilst negative selection is shown in light blue. Sites under significant positive selection in at least 50% of study participants are shown in purple. Variable loops V3, V4 and V5 are contained within the three boxes, respectively. The dotted lines denote 50% of study participants. Antibody epitope clustering is shown in grey, whereby intensity denotes number of epitopes spanning that residue as reported in the LANL Immunology Database (http://www.hiv.lanl.gov/content/immunology). Sequences have been aligned to the HXB2 Gp120 reference sequence (accession number K03455), and position is relative to this alignment. (**B)** Homology-modelled structure of the SM cohort consensus Gp120 sequence in surface representation. Variable loops V3, V4 and V5 are shown in grey and sites under significant positive selection in 50% or more study participants are shown in purple. Structure has been modelled on a glycosylated HIV-1 Gp120 trimer (RCSB PDB 3J5M)^74^. For clarity, two molecules in the trimer are shown in line representation in grey.

Within the variable loops, substantial negative selection could be seen in V3, but not in V4 and V5 (Fig. 2A). The negative selection in V3 corresponded with a marked increase in the density of neutralising antibody epitopes. Five codons, corresponding to positions T297, A337, S348, D415 and S468 in the HXB2 Gp120 (accession number K03455), were under significant positive selection in 50% or more of the study participants irrespective of HLA profile (Fig. 1 and Table 1). These sites were further mapped to a homology-modelled structure of the SM cohort Gp120 consensus, and clustered either within or immediately flanking the variable loops on the distal exposed edge of the protein complex (Fig. 2B).

### Significant selection pressure was exerted by the humoral response

**Figure 3:**
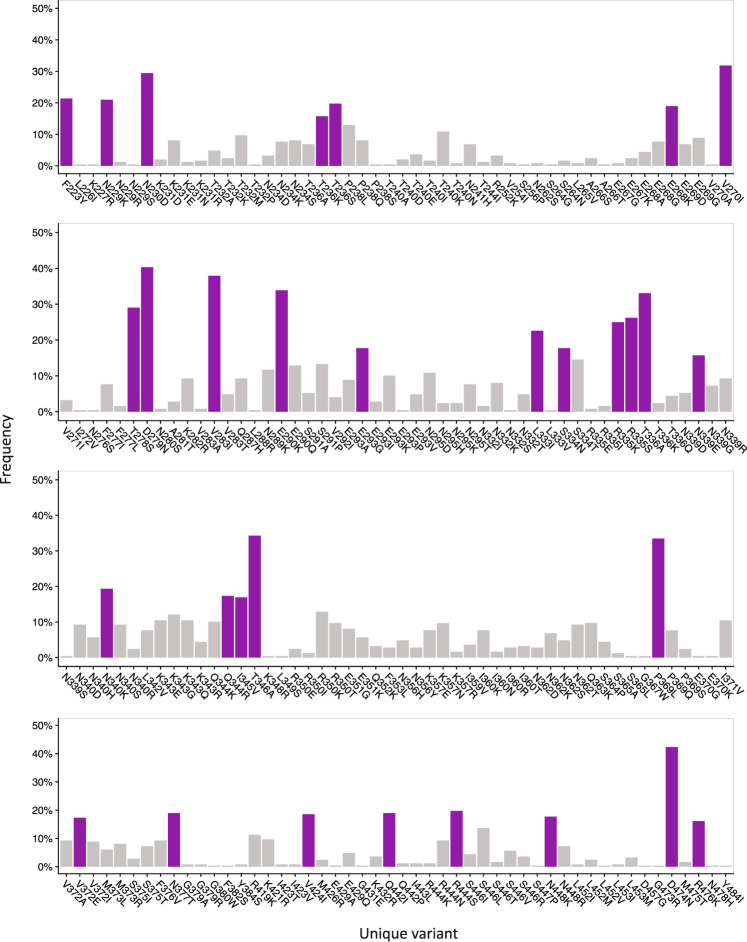
Mapping of variant frequencies. Unique variants identified in the SM cohort envelope Gp120 sequences. Major variants present in greater than 15% of the sequences are shown in purple whilst minor variants (<15%) are shown in grey. Position is relative to HXB2 Gp120 (accession number K03455).

We next considered how the virus evolves in response to significant positive selection pressure. For each position in the partial Gp120 sequence, deviations from the inferred founder were detected and 288 unique amino acid variants were recorded across 128 variable sites (Fig. 3). The remaining 51 positions were completely invariant (29%). Of the variants identified, many were present in a single or small collection of sequences and are likely of limited biological relevance. Major variants were therefore resolved, and to prevent overrepresentation by particular study participants, a single time-point was selected for each study participant (the selection aimed to get an equal distribution of samples from each year of cohort follow-up). Thirty-one major variants were detected in total, across 29 sites (Fig. 3).

**Figure 4:**
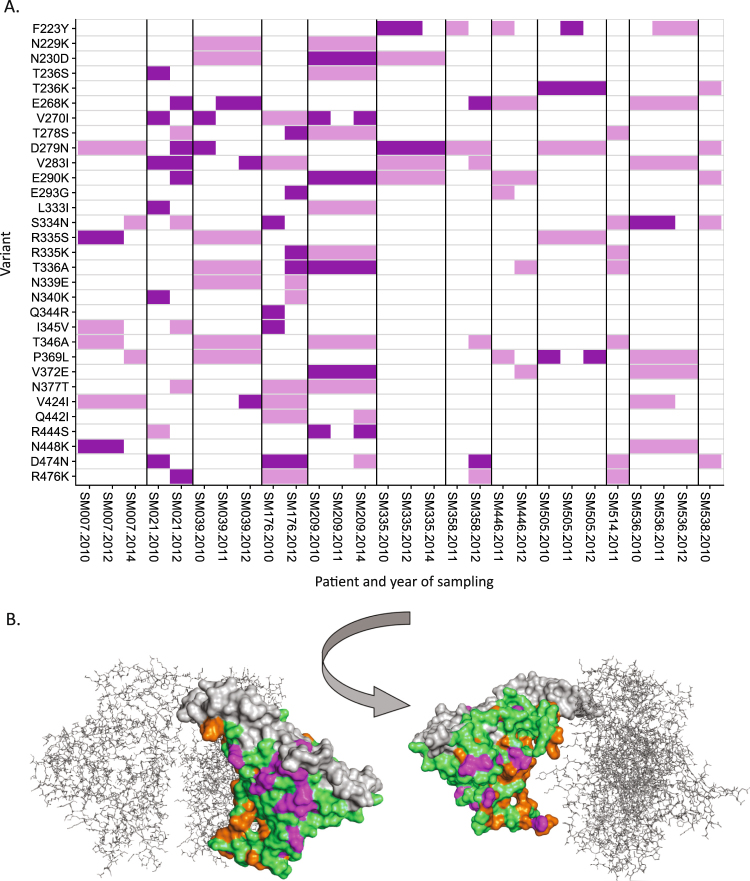
Overall presence of major variants. (**A**) Presence of major variants in individual study participants of the SM cohort at each time-point sampled. Those shown in dark purple are also under significant positive selection pressure within the specified individual, whilst those in light purple are either under significant negative selection or under no significant selection pressure. (**B)** Homology modelled structure of the SM cohort consensus Gp120 sequence, showing the variants under significant positive selection in 50% or more of the study participants in purple, and all invariant sites in orange. All variants except positions 345 and 424 are visible on the surface of the protein. Variable loops V3, V4 and V5 are shown in grey. Structure has been modelled on a glycosylated HIV-1 Gp120 trimer (RCSB PDB 3J5M)^74^. For clarity, two molecules in the trimer are shown in line representation in grey.

In study participants with longitudinally-sampled sequences, the presence or absence of each major variant was recorded at each time-point. Whether these sites were under significant positive selection pressure in that study participant was also determined (Fig. 4A). Twenty-four of the 29 sites housing major variants were under significant positive selection pressure in at least one study participant, and of these sites, a higher proportion exhibited fluctuating patterns of variant emergence than a consistent pattern wherein the variant was present at all time-points sampled (*p* < 0.01, two-tailed Fisher’s Exact Test).

Mapping these 24 sites to the homology-modelled structure of the SM cohort Gp120 consensus revealed that all but two were visible on the surface of the protein and likely accessible to antibodies (Fig. 4B). The exceptions were positions 345 and 424. Position 345 houses the major variant I345V, and is contained within the location of a known HLA-A11-restricted CTL epitope^42^. This position was found only to be under significant positive selection pressure in study participant SM176, who also expresses HLA-A11. Position 424 houses the CD4 binding site. The wholly invariant sites were similarly mapped, and the overwhelming majority clustered on the inner face of the quaternary structure.

### Despite significant antibody-mediated selection pressure, some sites housed a limited repertoire of amino acids

**Figure 5:**
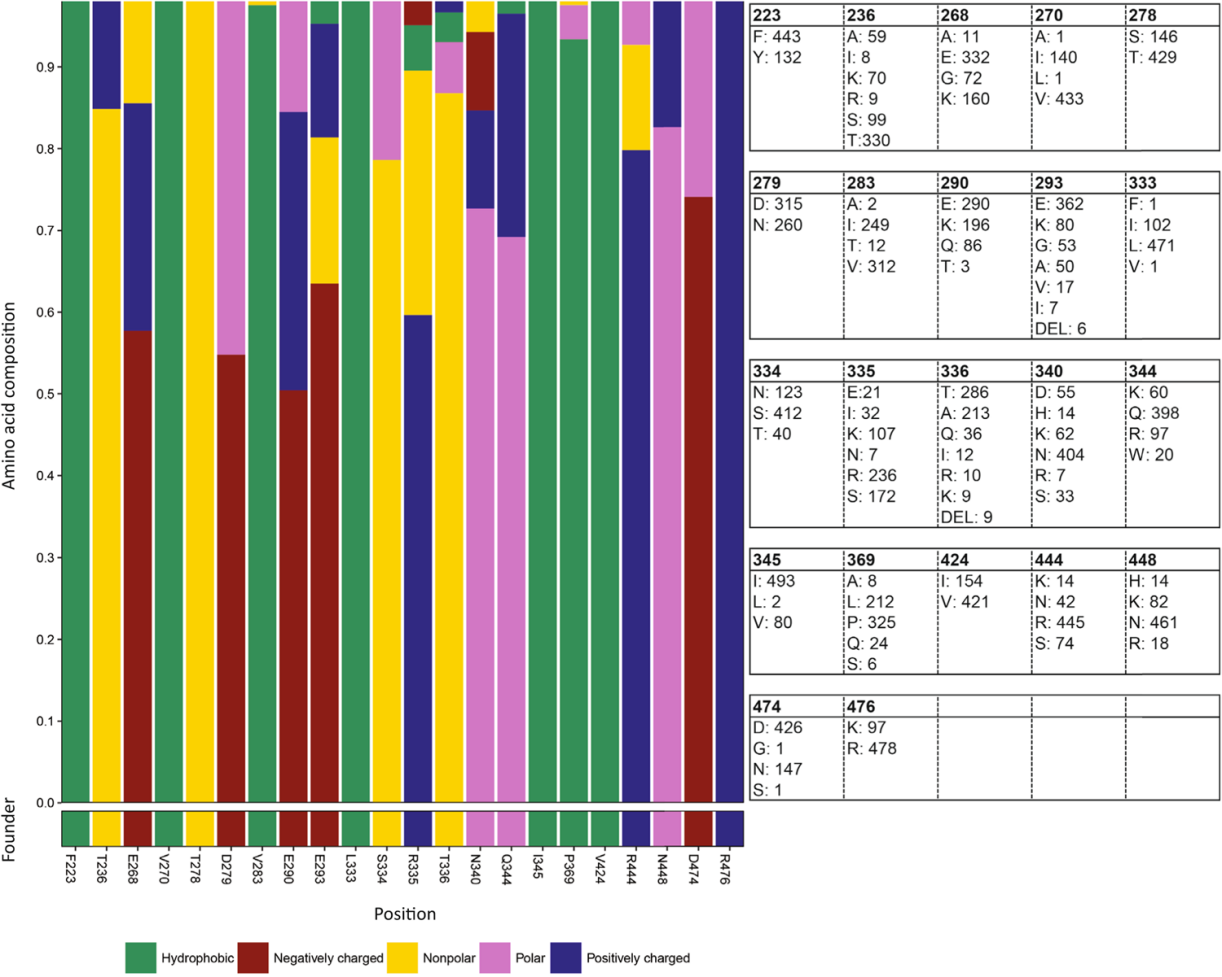
Biophysical properties of amino acids found in sites housing major variants. The biophysical properties of the amino acids found in each site, coloured according to the Lesk format^81^. The table show the composition of each position as the total number of each amino acid found at that site.

We next considered the biophysical diversity of amino acids in each of the 22 sites under significant positive selection pressure and visible on the surface of the protein (Fig. 5). In all positions, the biophysical properties of the amino acid in the inferred founder were preserved in approximately 50% or more of the sequences. Little divergence was seen in sites where the inferred founder residue was hydrophobic, with other properties being somewhat more variable. Tabulating the amino acids present in each site also reveals that seven positions flick back and forwards between just two or three amino acids.

### Four novel sites were identified and consistent with antibody activity

The 22 sites under significant positive selection pressure and visible on the surface of the protein were also cross-checked for existing antibody epitopes in the LANL Immunology Database and Genome Browser (Table 2). Twenty of these 22 sites had previously been associated with neutralising antibody activity, whereof 18 were part of previously described antibody epitopes from different sources (human, mice, or both). In detail, five positions (T236, Q344, I345, V424, and D474) were contained within known human antibody epitopes, whilst 16 positions were contained within epitopes reported in mice. Two sites flanking the V3 hypervariable loop (E290 and L333) – which itself houses the majority of antibody epitopes – were identified against which no antibodies have yet been reported. The L333 site primarily switched between I/L.

**Table 2:** Antibody epitope sequences corresponding to the sites identified on the surface of the HIV-1 Envelope. Data made available by the Los Alamos National Laboratory (LANL) Immunology Database (http://www.hiv.lanl.gov/content/immunology). Position is relative to HXB2 Gp120 (accession number K03455). Additional information about the neutralising antibody positions and associations can be found with references in the LANL HIV Genome Browser (https://www.hiv.lanl.gov/content/sequence/genome_browser/browser.html).

**Variant**	**Position**	**MAb ID** ^1^	**Sequence of antibody epitope**	**Source**	**Neutralising Antibody position** ^2^	**Neutralising antibody context**
F223Y	223	GV4H3	APAGFAIL	mouse	N	
		493-156	EPIPIHYCAPAGFAILKCNN	mouse	N	
		J1	GFAILKCNNK	mouse	N	
T236S	236	1006-30-D	KGSCKNVSTV	**human**	Y	This position has a strong co-variation with potency and structural proximity. This position is part of a common glycosylation site at position 234.
E268K	268	B12	RPVVSTQLLLNGSLAEEEVV	mouse	Y	This is an antibody b12 signature site in which E or S has been associated with sensitivity, and K or R has been associated with resistance. Other antibodies associated with this site include VRC01.
		110.E	NGSLAEEEVVIRSVNFTDNA	mouse	Y	
V270I	270	B12	RPVVSTQLLLNGSLAEEEVV	mouse	N	
		110.E	NGSLAEEEVVIRSVNFTDNA	mouse	N	
T278S	278	110.E	NGSLAEEEVVIRSVNFTDNA	mouse	Y	Antibodies associated with this site include: 1B2530, 3BNC60, 3BNC117, 8ANC131, 8ANC195, N6, NIH45–46, VRC01, VRC03, and VRC-PG04.
		110.C	VIRSVNFTDN	mouse	Y	
D279N	279	110.E	NGSLAEEEVVIRSVNFTDNA	mouse	Y	This site has been associated with the loss of transmitted-founder sequence, suggested to represent antibody-driven selection (Hraber *et al*.^82^). Antibodies associated with this site include: 1B2530, 3BNC55, 3BNC117, A16, CH103, CH235, N6, NIH45–46, VRC01, VRC03, VRC18, VRC27, and VRC-PG04.
		110.C	VIRSVNFTDN	mouse	Y	
V283I	283	NA^3^	NA^3^	NA^3^	Y	Antibodies associated with this site include: HJ16, N6, VRC01, VRC03, VRC13, VRC16, VRC27, and VRC-PG04.
E290K^4^	290	NA^3^	NA^3^	NA^3^	N	
E293G	293	IIIB-V3-26	SVEINCTRPNNNTRKSI	mouse	N	
L333I^4^	333	NA^3^	NA^3^	NA^3^	N	
S334N	334	NA^3^	NA^3^	NA^3^	Y	This site has been described as a supersite of vulnerability for antibody neutralisation^83^. The site has also been associated with the loss of transmitted-founder sequence, suggested to represent antibody-driven selection (Hraber *et al*.^82^). Antibodies associated with this site include: 2G12, PCDN33A, PCDN38A, and PCDN38B.
R335S/K	335	P1H6	SSNWKE	mouse	Y	Antibodies associated with this site include the PCDN antibodies.
T336A	336	P1H6	SSNWKE	mouse	N	
N340K	340	P1H6	SSNWKE	mouse	N	
		P3B2	WKEM(D/N)R	mouse	Y	Site shown to be under significant selection following 3BNC117 immunotherapy.
Q344R	344	P3B2	WKEM(D/N)R	mouse		
		838-D	KSITK	**human**		
		1006-15D	KSITKG	**human**		
		1027-15D	KSITKGP	**human**		
I345V	345	838-D	KSITK	**human**	N	
		1006-15D	KSITKG	**human**		
		1027-15D	KSITKGP	**human**		
P369L	369	4D7/4	IFKQSSGGDPEIVTHSFNCGG	mouse	Y	This is an antibody b12 signature site in which A or P has been associated with sensitivity, and I, L, or Q has been associated with resistance. Other antibodies associated with this site include: CH103, and IGg1b12.
		36.1(ARP 329)	FKQSSGGDPEIVTHSFNCGGE	mouse		
V424I	424	2D3	RIKQIINMWQEVGKAMYAPPI	mouse	N	
		JL413	KQIINMWQEVGKAMYA	**human**		
		5C2E5	QFINMWQEVK	mouse		
		G3-211	IINMWQKVGKAMYAP	mouse		
R444S	444	polyclonal	KAMYAPPISGQIRCSSNITG	mouse	Y	The S444 has been associated with increased susceptibility to neutralisation by 10-996.
N448K	448	polyclonal	KAMYAPPISGQIRCSSNITG	mouse	Y	Antibodies associated with this site include: 2G12, 3BC176, 3BC315, and PGT151-PGT158.
D474N	474	polyclonal	LTRDGGNNNNESEIFRPGGGD	**human**		Antibodies associated with this site include: 12A21, 8ANC131, 8ANC134, HJ16, N6, NIH45-46, VRC01, VRC03, VRC16, VRC27, and VRC-PG04.
		9201	GGGDMRDNWRSE	mouse		
		1C1	GGGDMRDNWRSELYKYKVVK	mouse	Y	
		H11	GGDMRD	mouse		
		W2	GGDMRDNWRSELYKYKVVKI	mouse		
D476K	476	9201	GGGDMRDNWRSE	mouse	Y	Antibodies associated with this site include: 8ANC131, 8ANC134, A16, N6, NIH45–46, VRC01, VRC27, and VRC-PG04.
		1C1	GGGDMRDNWRSELYKYKVVK	mouse		
		H11	GGDMRD	mouse		
		W2	GGDMRDNWRSELYKYKVVKI	mouse		

^1^Name of monoclonal antibody or “polyclonal” (if a general response is being studied) as listed in the LANL Immunology database.

^2^Y = Yes; N = No.

^3^NA = Not available.

^4^Novel site identified in the current study.

## Discussion

In line with previous observations^43^, mapping the ratio of non-synonymous to synonymous substitutions showed that the majority of sites within the C2-V5 region of Env Gp120 were under negative selection in all but one study participant out of ten. Whilst V4 and V5 loops exhibited a dearth of negative selection, the V3 hypervariable loop contained substantial negative selection. Of the five variable loops in Gp120, V3 is the most conserved with amino acid variation restricted to approximately 20% of the loop’s residues^44^. It is also likely that V3 is subject to stronger functional constraints due to its important role in co-receptor binding^45–48^. Moreover, it has been shown that deletion of V3 abrogates viral infectivity^49^.

Several sites within each study participant showed evidence of significant positive selection, and five of these were common to at least half of the study participants sampled. Structural modelling demonstrated that all but two of the 24 positively selected sites were found on exposed regions of the outer face of the protein complex. CTL epitopes in the HIV-1 Nef protein have been reported to cluster in hydrophobic regions^50^, whilst more recent evidence suggests that their distribution may be random across the genome^51^. Such strong clustering on the surface of the protein is therefore more consistent with antibody-mediated than CTL-mediated selection pressure^52^. The exceptions in terms of surface exposure were positions 345 and 424, which were buried within the protein. Notably, position 345 was found to be under significant positive selection pressure in only one study participant, SM176. This position is contained within a known HLA-A11-restricted CTL epitope, which is one of the HLA alleles expressed by study participant SM176. It is therefore feasible that this variant has emerged in response to CTL-mediated selection pressure in this study participant. Conversely, position 424 is important in CD4 binding^53^, and is contained within a known human antibody epitope. Mutation of this residue to methionine has been shown to increase susceptibility to neutralisation^54^.

We were able to assign most positions to known antibody epitopes in humans and mice. We also identified two novel sites, which were not contained within any known antibody epitopes reported in the literature. Position 290 has, however, been associated with the CTL-restricted epitope AKTIIVQLTEPVE in the HIV-1 CRF02_AG lineage (https://www.hiv.lanl.gov/content/sequence/genome_browser/browser.html). Moreover, these sites flank the V3 hypervariable loop, which is the most epitope dense region of Gp120 (LANL Immunology Database; https://www.hiv.lanl.gov/content/immunology/), although this may be due to a bias in reporting stemming from the extensive study of V3 in vaccine design rather than a genuine increase in immune activity. Further characterisation by neutralisation experiments could help to confirm these novel surface-exposed epitopes as targets by humoral or cellular immune responses.

Whilst numerous antibodies against V3 have been described, the cross-neutralisation potential of these antibodies is generally low, reviewed by Hartley *et al*.^55^. Glycosylation, sequence variation, masking by V1-V2, and the specific amino acid make-up of the loop may contribute to this, reviewed by Pantophlet *et al*.^56^. In addition, more complex causes of mutations, such as blockade of the accessibility of antibodies to the real epitopes or compensatory mutations to primary changes induced by antibodies, cannot be excluded. However, some monoclonal and polyclonal antibodies specific to epitopes within V3 have been demonstrated to neutralise diverse HIV-1 strains *in vitro*^57–60^. Two of the sites exhibit particularly limited amino acid diversity and as such may warrant further investigation as potential components of vaccines targeting shared epitopes of very low diversity within V3.

Indeed, despite evidence for significant antibody-mediated selection pressure, some sites were relatively conserved in terms of their composition, containing only a limited number of biophysically similar amino acids. This could be due to functional or structural constraints on the protein, and may reduce the ability of the virus to successfully escape antibodies targeting these regions. We also identified sites containing biophysically diverse amino acids that may be contained within epitopes eliciting effective antibody responses which cycle continuously between a limited number of biophysically distinct forms throughout chronic infection, as predicted by a previous modelling study^21^. Consistent with this model, we found evidence within individuals that some sites contain major variants that appear and disappear over time, such as proline to leucine in position 369 and arginine to serine in position 444.

In summary, our data provide insight into how and where the surface of Gp120 is mutating over the course of clinically latent infection. Our detailed analysis of HIV-1 evolution and selection allowed us to identify amino acid sites under positive selection that was likely attributed to host factors (since the study participants were infected with a purportedly identical founder virus). In line with this, it is also possible that site-specific selection may be virus-specific. Importantly, this comprehensive mapping resulted in the identification of both previously described and novel constrained antibody and T-cell epitopes. These sites may be crucial to viral envelope function, and if verified in functional studies, provide suitable targets for future drugs and vaccines.

## Methods

### Cohort characteristics and sampling

The SM cohort comprises HIV-1 infected study participants from a small rural community in Henan province, China, as described previously^38^. Between 1993 and 1995, the participants were infected with a narrow-source HIV-1 subtype B virus during a blood plasma donation scheme. Few individuals knew that they were infected until HIV screening programmes were implemented in China in 2004. Study participants were then recruited to the cohort.

Study participants with samples collected longitudinally over the course of HIV-1 infection permit detailed evolutionary analysis and identification of sites under selection. Twelve study participants from the SM cohort with multiple samples collected between 2010 and 2014 were available for this study (Table 1). All study participants gave informed consent for their samples to be used for research purposes.

### HIV-1 *env gp120* sequencing and sequence assembly

Viral RNA was isolated from cryopreserved plasma samples and purified using the QIAamp Viral RNA Extraction Kit (Qiagen) followed by reverse transcription using the SuperScript III Reverse Transcriptase System (Invitrogen). The *Env gp120* C2-V5 (approximately 799 bp, HXB2 [accession number: K03455] positions 6883–7681) was amplified by nested touchdown PCR (primers listed in Table S1). Amplified DNA was purified using the MinElute Gel Extraction Kit (Qiagen), and ligated into a pCR4-TOPO sequencing vector using the TOPO TA Cloning Kit for Sequencing (Invitrogen). Chemically competent One Shot MAX Efficiency DH5α *E*. *coli* (Invitrogen) were transformed with the prepared plasmids, and cultured overnight at 37 °C. Eighteen colonies were selected for colony PCR (M13F and M13R primers, Table S1), and the resulting products were purified using ExoSAP-IT (Affymetrix) and sequenced to generate forward and reverse reads (Source BioScience). Contigs were assembled and controlled manually using Geneious v9.0.5^61^ (HXB2 *gp120* positions 661–1455, accession number K03455). Sequences were multiple aligned using MUSCLE^62^, and then manually edited in MEGA v6.06^63^. Sequences were controlled for intra-patient clustering by maximum-likelihood phylogenetics.

### Inference of infecting founder strain

To infer the founder HIV-1 strain of the infected study participants, a consensus sequence was generated for each study participant for each time-point, with an ambiguity threshold of 10%. One sequence was selected per study participant to generate a dataset with sequences evenly distributed across the sampling period. The sequences were aligned and codon-stripped to a final alignment length of 759 nucleotides. Study participant SM007 was excluded from this analysis because it was not possible to conclusively rule out dual- or superinfection as preliminary data exploration demonstrated that sequences from this study participant did not resolve monophyletically.

Bayesian Markov Chain Monte Carlo phylogenetic inference - implemented through BEAST v1.8.2 (http://beast.bio.ed.ac.uk/beast/)^64^ - was used to estimate the time to the most recent common ancestor (tMRCA). Divergence time was estimated using the SRD06 model^65^, and an uncorrelated lognormal relaxed molecular clock^66^ with a rate prior of 0.001 substitutions per site per year. The Markov chain was run for 100,000,000 generations to allow for adequate mixing, with posterior samples extracted every 10,000 generations. A burn-in period of 10% was applied, and convergence of posterior probabilities was assumed once the effective sample size (ESS) of each parameter exceeded 200, as determined in Tracer v.1.5 (http://tree.bio.ed.ac.uk/software/tracer/). Three runs were combined in LogCombiner v1.8.2 (http://beast.bio.ed.ac.uk/LogCombiner/) and the mean root height of the tree was calculated. Phylogenetic trees were annotated in FigTree v.1.4.2 (http://beast.bio.ed.ac.uk/figtree).

As the SM cohort study participants were infected with a narrow source of virus, the sequence of the MRCA was used as a surrogate for the infecting founder. The sequence of the reconstructed ancestor at the tree root from each run following burn-in was extracted, and a consensus sequence was generated from an alignment of these sequences (26,000 sequences) with an ambiguity threshold of 10%.

### Viral subtyping

An unambiguous consensus sequence was generated from the sequences of each time-point for each study participant. The sequences were then aligned to the LANL 2005 *gp120* reference dataset (http://www.hiv.lanl.gov/), and viral subtyping was performed by Bayesian phylogenetic inference (BEAST v1.8.2). The generalised time-reversible (GTR) nucleotide substitution model plus invariant sites and gamma-distributed rate heterogeneity (GTR + I + G) was used, with a constant size coalescent tree prior, estimated base frequencies, and a strict molecular clock. Following exclusion of burn-in, TreeAnnotator v1.8.2 was used to determine the maximum clade credibility tree (MCC).

### Selection pressure in *env gp120*

The ratio of non-synonymous (dN) to synonymous (dS) mutations was estimated for each codon in study participant-specific alignments. Renaissance counting^41,67^ was implemented through BEAST v1.8.2, and the HKY85 nucleotide substitution model^68^, three-site codon partitioning, a strict molecular clock with tip-dating of time-stamped sequences were applied. Significant selection was defined as a 95% higher posterior density (HPD) range that did not encompass 1. An alignment representing all study participants was then constructed from these data, and the dN/dS estimates were combined across each aligned position. The proportion of study participants with virus showing evidence of significant selection pressure in each cohort was calculated.

### Variant characterisation

A variant was defined as any amino acid in any position in the alignment that differed from that present in the inferred founder. Owing to the extensive degree of variation, the hypervariable loops were conservatively stripped from the alignment prior to analysis (final length 179 amino acids). Major variants were defined as variants found in greater than 15% of the sequences. Whilst major variants are canonically defined as those present at a frequency greater than 5%, this value was conservatively tripled as the amplicon was approximately three times more variable than the full-length HIV-1 genome^69^.

### Structural modelling

Homology modelling was implemented through SWISS-MODEL^70–73^ to map the translated SM cohort Gp120 consensus sequence to a cryo-electron microscopy (cryo-EM) crystal structure of a glycosylated HIV-1 envelope trimer (RCSB PDB 3J5M)^74^. The sites of interest were annotated on the modelled structure in PyMOL v1.7.4 (Schrödinger, LLC.).

### Data analysis

Epitope mapping, selection mapping, variant characterisation, biophysical properties, statistical analysis, and plotting were all performed in R (v3.2.3)^75^ via the RStudio (v.0.98.1103) integrated development environment (http://www.rstudio.com/). The following libraries were used: dplyr^76^; scales^77^; gridExtra^78^; ggplot2^79^; reshape2^80^.

### Ethics Approval And Consent To Participate

Ethical approval was obtained for this study from Beijing You’an Hospital and the University of Oxford Tropical Ethics Committee (OxTREC).

### Availability of Data and Materials

The datasets generated and analysed during the current study are available in the Genbank repository (accession numbers: MF078678-MF079252). Custom R scripts used in the analysis of these data are available from the authors on request.

## Electronic supplementary material

Supplementary Information
