# Supplementary material for: Analysis of HIV-1 envelope evolution suggests antibody-mediated selection of common epitopes among Chinese former plasma donors from a narrow-source outbreak
Source: Sci Rep. 2018 Apr 10;8:5743. doi: 10.1038/s41598-018-23913-2 (PMC5893620; doi:10.1038/s41598-018-23913-2)
Supplement: Supplementary file 1 — Supplementary Information [file 41598_2018_23913_MOESM1_ESM.pdf]

Analysis of HIV-1 envelope evolution suggests antibody-mediated selection of common epitopes among Chinese former plasma donors from a narrow-source outbreak

Sophie M. Andrews[1], Yonghong Zhang[2], Tao Dong[3], Sarah L. Rowland-Jones[1#], Sunetra Gupta[4#], Joakim Esbjörnsson[1,5#*]

[1]Nuffield Department of Medicine, University of Oxford, Oxford, United Kingdom.

[2]Beijing You'an Hospital, Capital Medical University, Beijing, China.

[3]Weatherall Institute of Molecular Medicine, University of Oxford, Oxford, United Kingdom.

[4]Department of Zoology, University of Oxford, Oxford, United Kingdom.

[5]Department of Laboratory Medicine, Lund University, Sweden.

[*]Corresponding author and to whom requests for reprints should be made:
Nuffield Department of Medicine, University of Oxford, NDMRB, Old Road Campus, Roosevelt Drive, Headington, Oxfordshire, United Kingdom, OX3 7FZ. E-mail: joakim.esbjornsson@ndm.ox.ac.uk

[#]These authors contributed equally to this study.

SUPPLEMENTARY INFORMATION

Supplementary Figures

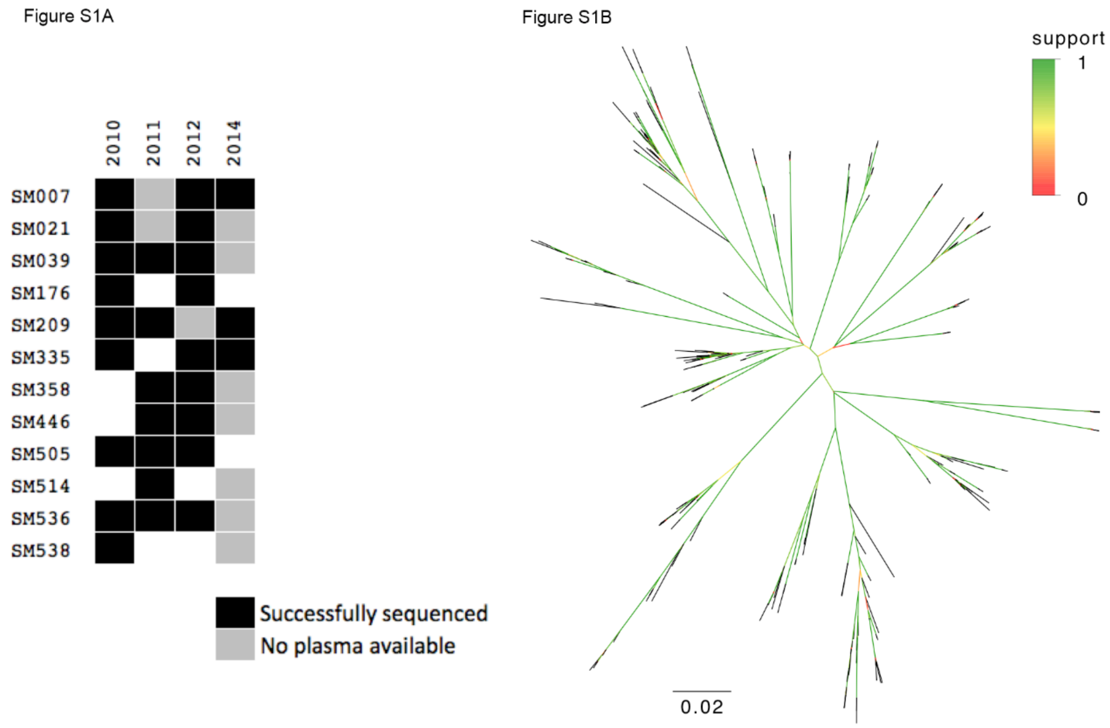

Figure S1C

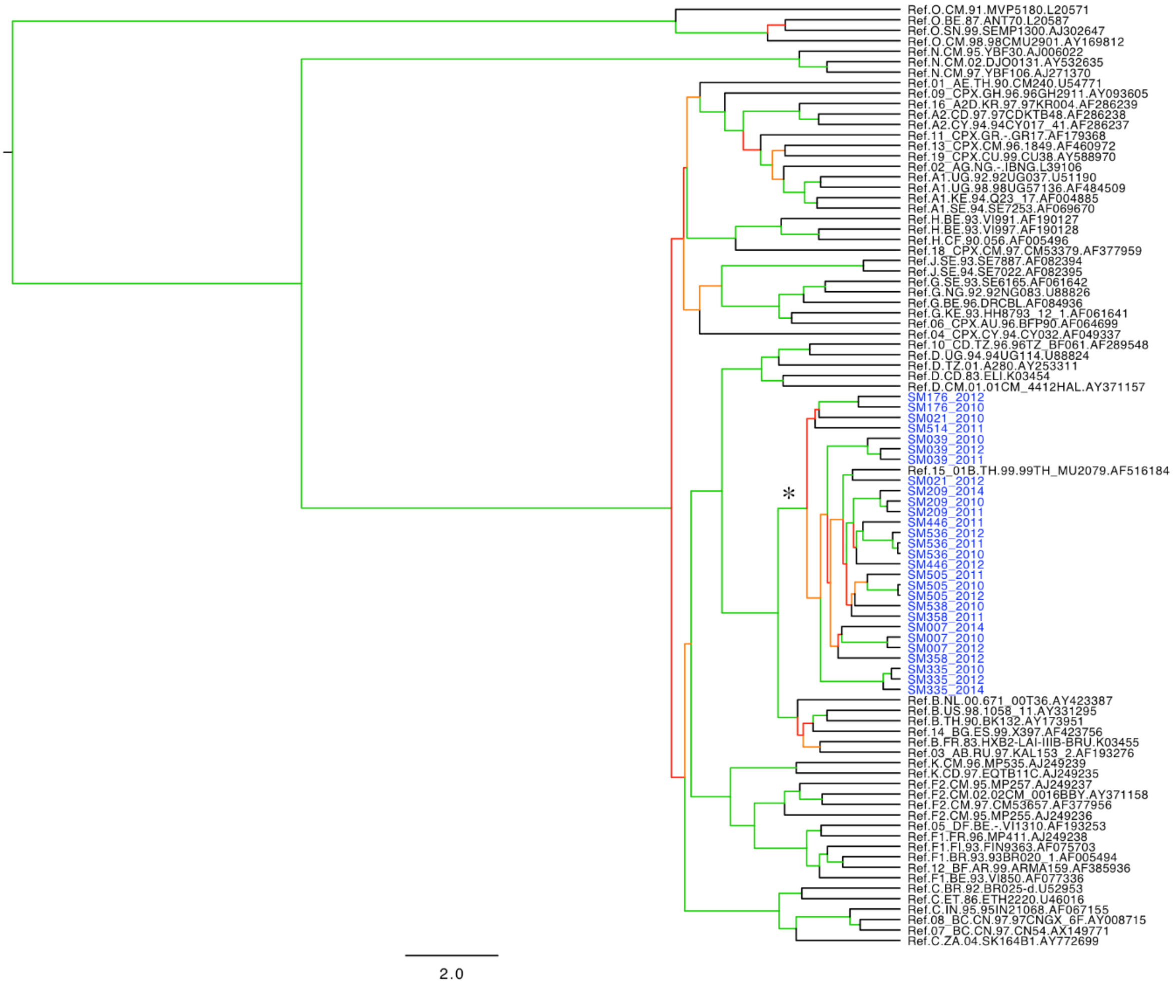
Ref.O.CM.91.MVP5180.L20571
Ref.O.BE.87.ANT70.L20587
Ref.O.SN.99.SEMP1300.AJ302647
Ref.O.CM.98.98CMU2901.AY169812
Ref.N.CM.95.YBF30.AJ006022
Ref.N.CM.02.DJO0131.AY532635
Ref.N.CM.97.YBF106.AJ271370
Ref.01_AE.TH.90.CM240.U54771
Ref.09_CPX.GH.96.96GH2911.AY093605
Ref.16_A2D.KR.97.97KR004.AF286239
Ref.A2.CD.97.97CDKTB48.AF286238
Ref.A2.CY.94.94CY017_41.AF286237
Ref.11_CPX.GR.-.GR17.AF179368
Ref.13_CPX.CM.96.1849.AF460972
Ref.19_CPX.CU.99.CU38.AY588970
Ref.02_AG.NG.-.IBNG.L39106
Ref.A1.UG.92.92UG037.U51190
Ref.A1.UG.98.98UG57136.AF484509
Ref.A1.KE.94.Q23_17.AF004885
Ref.A1.SE.94.SE7253.AF069670
Ref.H.BE.93.VI991.AF190127
Ref.H.BE.93.VI997.AF190128
Ref.H.CF.90.056.AF005496
Ref.18_CPX.CM.97.CM53379.AF377959
Ref.J.SE.93.SE7887.AF082394
Ref.J.SE.94.SE7022.AF082395
Ref.G.SE.93.SE6165.AF061642
Ref.G.NG.92.92NG083.U88826
Ref.G.BE.96.DRCBL.AF084936
Ref.G.KE.93.HH8793_12_1.AF061641
Ref.06_CPX.AU.96.BFP90.AF064699
Ref.04_CPX.CY.94.CY032.AF049337
Ref.10_CD.TZ.96.96TZ_BF061.AF289548
Ref.D.UG.94.94UG114.U88824
Ref.D.TZ.01.A280.AY253311
Ref.D.CD.83.ELI.K03454
Ref.D.CM.01.01CM_4412HAL.AY371157
SM176_2012
SM176_2010
SM021_2010
SM514_2011
SM039_2010
SM039_2012
SM039_2011
Ref.15_01B.TH.99.99TH_MU2079.AF516184
SM021_2012
SM209_2014
SM209_2010
SM209_2011
SM446_2011
SM536_2012
SM536_2011
SM536_2010
SM446_2012
SM505_2011
SM505_2010
SM505_2012
SM538_2010
SM358_2011
SM007_2014
SM007_2010
SM007_2012
SM358_2012
SM335_2010
SM335_2012
SM335_2014
Ref.B.NL.00.671_00T36.AY423387
Ref.B.US.98.1058_11.AY331295
Ref.B.TH.90.BK132.AY173951
Ref.14_BG.ES.99.X397.AF423756
Ref.B.FR.83.HXB2-LAI-IIIB-BRU.K03455
Ref.03_AB.RU.97.KAL153_2.AF193276
Ref.K.CM.96.MP535.AJ249239
Ref.K.CD.97.EQTB11C.AJ249235
Ref.F2.CM.95.MP257.AJ249237
Ref.F2.CM.02.02CM_0016BBY.AY371158
Ref.F2.CM.97.CM53657.AF377956
Ref.F2.CM.95.MP255.AJ249236
Ref.05_DF.BE.-.VI1310.AF193253
Ref.F1.FR.96.MP411.AJ249238
Ref.F1.FI.93.FIN9363.AF075703
Ref.F1.BR.93.93BR020_1.AF005494
Ref.12_BF.AR.99.ARMA159.AF385936
Ref.F1.BE.93.VI850.AF077336
Ref.C.BR.92.BR025-d.U52953
Ref.C.ET.86.ETH2220.U46016
Ref.C.IN.95.95IN21068.AF067155
Ref.08_BC.CN.97.97CNGX_6F.AY008715
Ref.07_BC.CN.97.CN54.AX149771
Ref.C.ZA.04.SK164B1.AY772699
*
2.0

Supplementary Figure legends

Figure S1. Cohort characterisation. A) PCR sequencing success for the samples available. Successfully sequenced samples are shown in black, and failed samples are shown in white. Samples shown in grey were unavailable. **B)** Maximum likelihood phylogenetic tree of sequences obtained. Branch support is indicated by colour, as defined by the internal key. The star-like shape of the phylogeny with short internal branches supports the narrow-source origin of the cohort.

Figure S2. Subtyping. Phylogenetic subtyping of SM cohort viral sequences represented as a midpoint-routed maximum clade credibility (MCC) tree of intrapatient consensus sequences (blue) aligned to the 2005 LANL *gp120* reference dataset (black). Branch support: ≥ 0.95 green; ≥ 0.75 orange; < 0.75 red. Support for the node associating all SM cohort sequences with CRF 15-01B (marked with an asterisk) is 1.

Supplementary Tables

Table S1. Primers used in the amplification and sequencing of the HIV-1 *env gp120* fragment.

Name	Sequence	Target	Corresponding to HIV-1 HXB2 (K03455)
FPD_Env_OF	AAAGAGCAGAAGACAGTGGCAATG	HIV-1 gp120	HXB2 6204-6227
FPD_ENV_OR	AGCCTCCTACTAYCATTATGAAT	HIV-1 gp120	HXB2 8276-8298
FPD_ENV_IF	CGGCTGGTTTTGCGATTCTA	HIV-1 gp120	HXB2 6883-6902
FPD_ENV_IR	TATTTATATAATTCACTTCTCCA	HIV-1 gp120	HXB2 7659-7681
M13F	GTAAAACGACGGCCAG	pCR®4-TOPO	355-370
M13R	CAGGAAACAGCTATGAC	pCR®4-TOPO	205-221
